# Undertreatment or Overtreatment With Statins: Where Are We?

**DOI:** 10.3389/fcvm.2022.808712

**Published:** 2022-04-29

**Authors:** Irene García-Fernández-Bravo, Ana Torres-Do-Rego, Antonio López-Farré, Francisco Galeano-Valle, Pablo Demelo-Rodriguez, Luis A. Alvarez-Sala-Walther

**Affiliations:** ^1^Internal Medicine, Hospital General Universitario Gregorio Marañón, Madrid, Spain; ^2^Grupo (departamento) de investigación Riesgo cardiovascular y lípidos, Instituto de investigación Sanitaria Gregorio Marañón (IiSGM), Madrid, Spain; ^3^Department of Medicine, School of Medicine, Universidad Complutense de Madrid, Madrid, Spain

**Keywords:** statins, cardiovascular risk, HDL-cholesterol, LDL-cholesterol, PCSK9 inhibitor, primary prevention, secondary prevention after myocardial infarction

## Abstract

Statins, in addition to healthy lifestyle interventions, are the cornerstone of lipid-lowering therapy. Other low-density lipoprotein (LDL)-lowering drugs include ezetimibe, bile acid sequestrants, and PCSK9 inhibitors. As new evidence emerges from new clinical trials, therapeutic goals change, leading to renewed clinical guidelines. Nowadays, LDL goals are getting lower, leading to the “lower is better” paradigm in LDL-cholesterol (LDL-C) management. Several observational studies have shown that LDL-C control in real life is suboptimal in both primary and secondary preventions. It is critical to enhance the adherence to guideline recommendations through shared decision-making between clinicians and patients, with patient engagement in selecting interventions based on individual values, preferences, and associated conditions and comorbidities. This narrative review summarizes the evidence regarding the benefits of lipid-lowering drugs in reducing cardiovascular events, the pleiotropic effect of statins, real-world data on overtreatment and undertreatment of lipid-lowering therapies, and the changing LDL-C in targets in the clinical guidelines of dyslipidemias over the years.

## Introduction

Clinical trials have been the way to define the real role of new drugs in clinical practice, demonstrating their safety, efficacy, and mainly answering the key question: does this drug represent a clinical benefit with better reductions in cardiovascular risk, becoming a real advance compared to the previous options?

As new evidence from new clinical trials emerges, therapeutic goals change, giving rise to renewed clinical guidelines. However, some clinicians show reluctance to periodically review new guidelines, since it means upgrading their approaches and changing their concepts.

Cardiovascular diseases are among the two leading causes of morbidity and mortality in Western countries. A conservative attitude, without updating the knowledge of the latest cardiovascular prevention trials, means limiting the population who could potentially benefit from these treatments.

Currently, in patients with a very high cardiovascular risk, there is no clear cut point to consider safety below it, which leads to the paradigm in the reduction of low-density lipoprotein cholesterol (LDL-C) that “the lower, the better” (1, 2). Probably, the cost/benefit analysis will help determine what LDL-C levels should be achieved in these patients.

This narrative review summarizes the evidence regarding the benefits of lipid-lowering drugs in reducing cardiovascular events, the pleiotropic effect of statins, real-world data on overtreatment and undertreatment of lipid-lowering therapies, and the changing LDL-C in targets in the clinical guidelines of dyslipidemias over the years.

## Pleiotropic Effects of Statins

**Figure 1:**
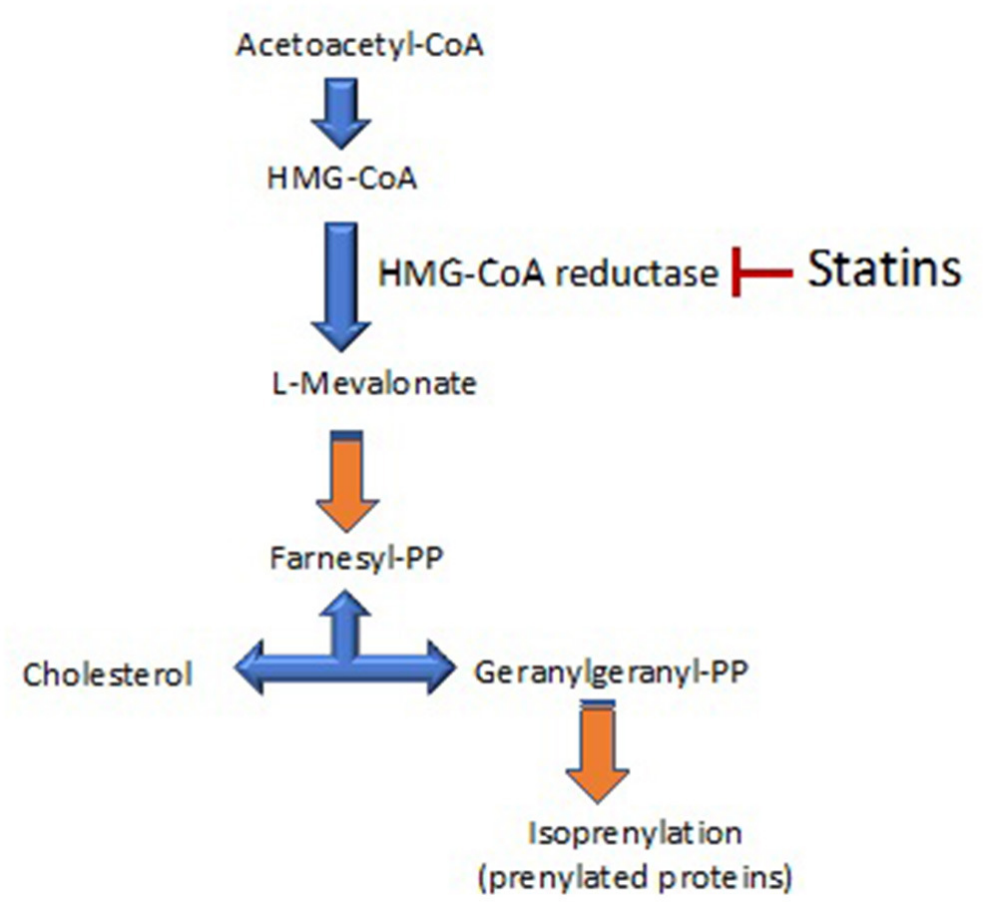
A scheme of the cholesterol synthesis pathway. 3-hydroxy-3-methyl-glutaryl-coenzyme A reductase (HMG-CoA reductase) is the rate-controlling enzyme of the mevalonate pathway involved in cholesterol synthesis. This pathway also produces the biosynthesis of isoprenoids. The main target of statins is the inhibition of HMG-CoA reductase.

“Pleiotropy” has its origin in the Greek words “pleion,” which means more, and “tropia,” which means response or stimulus. The pleiotropic effects of a drug are actions other than those for which the agent was specifically developed. In the case of statins, their pleiotropic effects could then be defined as the effects of statins that are not dependent of changes in cholesterol levels. The main pleiotropic effects of statins are exerted by inhibiting the enzyme HMG-CoA reductase (3-hydroxy-3-methyl-glutaryl-coenzyme A reductase), which converts HMG-CoA to L-mevalonate. The lack of generation of L- inhibits the formation of isoprenoids, such as farnesylpyrophosphate (FPP) and geranylgeranylpyrophosphate (GGPP) (Figure 1).

The cardiovascular benefits of statins have been conventionally attributed to reduction of LDL-C. However, in trying to explain some clinical benefits of statins, subanalyses of large clinical trials have also suggested direct cardioprotective effects of this family of drugs. To give an example, the Anglo-Scandinavian Cardiac Outcomes Trial (ASCOT) was a multicenter randomized trial initially designed to compare two anti-hypertensive treatment strategies for the prevention of coronary heart disease (CHD) in hypertensive patients who had no history of CHD. That trial included a double-blind, randomized comparison of the cardiovascular effects of a statin, atorvastatin, with placebo among patients who had mild-moderate high total cholesterol (TC) concentrations ( ≤ 250 mg/dL) (3). The ASCOT trial demonstrated relative risk reduction for CHD, fatal and non-fatal stroke, total cardiovascular events, and total coronary events with statin therapy, regardless of baseline lipid values. In fact, baseline TC levels were not significantly different between the atorvastatin group and the placebo group (3). Another example of the possible clinical pleiotropic effect of statins is The West of Scotland Coronary Prevention Study (WOSCOPS trial), where time-to-event curves began to diverge within 6 months of initiation of therapy, an effect which evidently occurred earlier than that predicted from cholesterol decline alone (4).

Based on these and other trials, it has been speculated that statins exert some of their clinical cardiovascular benefits beyond their lipid-lowering property. Several mechanisms have been proposed to explain the beneficial cardiovascular effects of statins. Among them, the improvement of endothelial function, reduction of the inflammatory response, and mitochondrial effects stand out. However, statins also contribute to undesirable effects, including insulin resistance, type-2 diabetes, and pro-calcifying effects.

### Endothelial Functionality

It is well-established that statins have beneficial effects on the vascular endothelium and improve endothelial functionality. In fact, statins could upregulate endothelial nitric oxide synthase (NOS3), the enzyme that forms nitric oxide in the endothelium. One of the ways that statins modulate NOS3 is by reducing the Rho/Rock signaling, which increases the stability of NOS3 mRNA and thus enhances NOS3 expression (5). Interestingly, some of the main targets of statins are Ras and Rho proteins. In endothelial cells, translocation of Ras from the cytoplasm to the plasma membrane requires farnesylation, whereas translocation of Rho requires gerenal geranylation. Upward expression of NOS3 protein by statins appears to depend on inhibition of isoprenylation. Inhibition of isoprenylation leads to cytoplasmic accumulation of inactive forms of Ras and Rho, resulting in upregulated expression of NOS3.

Another way in which statins can promote NOS3 functionality is by acting on caveolin-1. The plasma membrane has regions called caveolae where cholesterol is the main component and where caveolin-1 is located. Caveolin-1 gene transcription is regulated by cholesterol-sensitive elements, and statins downregulate caveolin-1 synthesis. Downregulation of caveolin-1 promotes Ca + 2/calmodulin dissociation that induces NOS3 activity (6).

In cellular studies, inhibition of HMG-CoA reductase resulted in upregulation of endothelial NO synthase activity, resulting in increased bioavailability of NO, an important regulator of vascular tone, platelet aggregation, and the proliferation of vascular SMCs (7, 8). The latter is a key driver of atherosclerotic plaque progression, and statins have been shown to reduce vascular SMC proliferation and migration. This process is especially evident in the heart transplant population.

Statins have also been implicated in reducing platelet aggregation, in addition to having antithrombotic properties that may contribute to the overall reduction in cardiovascular death (7).

In cardiovascular diseases and, more particularly, in heart diseases, angiogenesis (the growth of vessels of small capillary size) and vasculogenesis (referring to the process in which progenitor cells form vascular structures *de novo*) are essential phenomena for recovery after tissue damage. In fact, recovery after ischemia or infarction in any organ requires the growth of blood vessels. The initiation of angiogenesis and the formation of early vascular structures seems to depend on circulating endothelial progenitor cells (9). Indeed, the number and functional capacity of circulating vascular or endothelial progenitor cells appear to influence both angiogenesis and vasculogenesis. It could be critical for some specific populations, such as the elderly and diabetic patients, in whom the number of circulating endothelial progenitor cells has been widely shown to be drastically reduced. In this sense, the incidence of stroke, claudication, and ischemia or myocardial infarction increases in both diabetic and elderly patients, and they have worse when ischemia or infarction occurs. Interestingly, hypercholesterolemia also leads to impaired angiogenesis (10).

As mentioned, several years ago, circulating undifferentiated cells derived from pluripotent stem cells, called endothelial progenitor cells (EPC), were identified. EPC can migrate to sites of neovascularization and then differentiate into endothelial cells. Statins promote the proliferation, migration, and survival of endothelial cells and bone marrow-derived EPC by stimulating the serine/threonine protein kinase Akt (also known as protein kinase B) pathway.

Two types of EPC have been identified and have been classified as early and late EPCs. Early EPCs were found to form elongated cells, while the late EPC population gave rise to cobblestone-like colonies with strong proliferation. Patients receiving statin therapy have increased number of late EPC (11).

### Statins and Anti-Inflammation

Inflammation as a major factor in atherogenesis seemed an intense investigation. Currently, there are many reports associating cytokines, cell adhesive molecules, metalloproteases, and other inflammatory-related molecules with the genesis and progression of cardiovascular disease. Cell culture experiments, animal models, and clinical trials have all supported the primary involvement of inflammation in cardiovascular disease. The anti-inflammatory properties of statins are probably their most widely analyzed and recognized pleiotropic effect. In this regard, several clinical studies have reported that, regardless of their lipid-lowering effects, statins reduce the levels of circulating pro-inflammatory molecules, such as levels of high-sensitivity C-reactive protein (hs-CRP). In addition, reduction of hs-CRP levels associated with statins has been shown in patients with and without established cardiovascular disease.

In the atherosclerotic plaque, statins decrease inflammation by promoting plaque stability through combined reduction of lipids, macrophage, and metalloproteinase activities. Some of these effects are due to the reduction of upstream mediators of cholesterol biosynthesis in the mevalonate pathway and, consequently, produce a reduction in protein prenylation that can affect the immune response (12).

Using proteomics, our group also analyzed possible changes in the map of proteins related to inflammation in the plasma of moderate hypercholesterolemic patients treated for 12 weeks with simvastatin (40 mg/day). Simvastatin treatment modified the plasma content of several proteins, but, specifically, the changes observed in the levels of apolipoprotein A-IV and haptoglobin isoform two did not correlate with reduction in plasma total cholesterol levels (13).

Knowledge of the pleiotropic effects of statins, including anti-inflammatory and immunomodulatory effects, has been increasing recently. In this regard, a series of reports have suggested the inhibition of toll-like receptors (TLR) by statins. TLRs are a class of transmembrane receptors that act as sentinels of both innate and adaptive immunity. Statins through inhibition of TLR4 expression and regulation of the TLR4/Myd88/NF-κB signaling pathway can slow the progression of atherosclerosis. TLR4 was further identified as the signaling receptor for E. coli lipopolysaccharide (LPS). Interestingly, statins inhibited LPS-induced activation of human peripheral mononuclear cells and endothelial cells (14). One study demonstrated the ability of statins to reduce the surface expression of TLR4 on CD14 + monocytes. The reduction in TLR4 expression followed a dose-dependent pattern and was reversed by GGPP (14–17).

Clinical research aimed to separate the LDL-C lowering benefit of statins from their potential pleiotropic effects in clinical trials has been hampered by the strong association between elevated cholesterol and CHD [18]. Studies comparing the effect of statin-mediated LDL-C lowering with an equal LDL-C lowering mediated by another intervention (e.g., diet) have reported pleiotropic effects of statins in animals (18). Nonstatins (e.g., ezetimibe) have been used in humans for this purpose. Ezetimibe lowers LDL-C by 15–20% and can only be compared to less potent statins, making vascular effects more difficult to observe (19).

PCSK9 inhibitors, a group of drugs that have recently appeared, target a protein (PCSK9) involved in the control of the LDL-C receptor (1, 2). PCSK9 antibodies studies have shown a decrease in cardiovascular risk by reaching a very low LDL-C concentration even in those patients with LDL-C close to 100 mg/dL, playing a role in changing LDL-C targets. Currently, the only approved PCSK9 inhibitors are two fully human mAbs, alirocumab, and evolocumab (1, 2). They demonstrated a decrease in the incidence of a composite of death from coronary heart disease, NMI, fatal or nonfatal ischemic stroke, or unstable angina, requiring hospitalization by reaching an LDL-C goal of 66 mg/dL (1) with alirocumab and close to 30 mg/dL with evolocumab (2) 48 months after randomization.

The notion of whether statin pleiotropy has clinical relevance in terms of cardiovascular risk reduction may benefit from ongoing trials with the PCSK9 inhibitors that could be compared with a high-dose statin in terms of LDL-C-lowering equivalence. High-dose statin therapy has shown impressive benefits in plaque reduction that has been postulated to be due to its anti-inflammatory effects in addition to its intensive LDL-C-lowering effects (1, 2). The results of the GLAGOV (GLobal Assessment of Plaque reGression with a PCSK9 antibOdy as Measured by intraVascular Ultrasound) study showed that the addition of PCSK9i to statins did not reduce plaque size, supporting the idea that plaque regression may be mainly due to pleiotropic effects of statins (20).

The FOURIER (21), ODYSSEY (22), SPIRE-1, and SPIRE-2 (23) studies are event-driven trials that have evaluated the effects of PCSK9 inhibitors on cardiovascular outcomes. Interestingly, a *post hoc* analysis of ODYSSEY found that, in patients treated with alirocumab, absolute reduction in lipoprotein (a) predicted absolute reduction in risk of a first event, regardless of the concurrent reduction of LDL-C (22). On the other hand, in a *post hoc* analysis of the SPIRE trials, PCSK9 inhibition with bococizumab had no effect on subclinical inflammation (as measured by hsCRP levels). Despite aggressive lowering of LDL-C, there was a continuous gradient in the risk of future cardiovascular events based on hsCRP (23). PCSK9i lowers LDL-C by a mechanism similar to statins in that they increase the LDL receptor-mediated hepatic uptake of ApoB-containing lipoproteins. However, they do not inhibit the mevalonate pathway, and would not have similar pleiotropic effects derived from Rho GTPase inhibition. Despite their potent LDL-C-lowering effects, PCSK9i does not reduce serum markers of inflammation (23). These observations do not exclude anti-inflammatory effects on circulating monocytes or vascular cells. Four large trials have recently evaluated the effects of anti-inflammatory drugs in the secondary prevention of major cardiovascular events (MACE) in more than 25,000 patients followed for 1.9–3.7 years (24–28). In summary, canakinumab and colchicine have shown efficacy in preventing MACE in patients with ischemic heart disease, but only colchicine has acceptable safety (and cost) for use in secondary cardiovascular prevention. Along with these trials, the PCSK9i outcome trials have provided important information insights into the relevance of reduction in systemic inflammatory markers to clinical outcomes.

### Mitochondrial Functionality and Statins

The main function of mitochondria is to synthesize ≥95% of adenosine triphosphate *via* the mitochondrial respiratory chain. However, they participate in many other functions, for example, they contribute to calcium handling, regulation of apoptosis, and the state of oxidative stress. In this regard, mitochondria are probably the main cellular source of reactive oxygen generation (ROS), but they also have a powerful anti-ROS machinery, including Mn-superoxide dismutase (superoxide dismutase isoform type 2). Mitochondria have a dynamic life based on several steps that maintain the number of mitochondria during cell division and the health of the mitochondria. This dynamic process called mitochondria biogenesis includes different steps: (a) the fusion of two mitochondria into one called mitochondrion; (b) fission or division of a single mitochondrion into smaller mitochondria, and (c) mitophagy, a specialized form of autophagy whose goal is to degrade dysfunctional mitochondria while maintaining the functional mitochondria population. This latter process is also associated with cell apoptosis (29).

The effects of statin on apoptosis have attracted attention mainly as a pharmacological tool for the treatment of certain tumors. In this sense, retrospective studies have reported a decrease in the cancer-specific mortality rate in patients treated with statins. In many cases, it was attributed to the apoptosis-inducing effect of statins. In addition, GGPP appears to be critical to inhibiting statin-induced apoptosis, suggesting that GGPP depletion by statins might be associated with impaired isoprenylation of proteins required for tumor cell growth (30, 31). However, the evidence on this effect is still scarce.

In the field of cardiovascular diseases, the effect of statins on apoptosis is not entirely clear. For example, a recent study has demonstrated that rosuvastatin inhibited apoptosis in human coronary artery endothelial cells through a decrease in mitochondrial ROS production, inflammation, and mitochondrial damage (32). The effect of rosuvastatin on mitochondria was mediated by the JAK2/STAT3 signaling pathway (32). However, other studies do not support the idea that statins are linked to a proapoptotic process. For instance, pravastatin increased *in vitro* expression of the pro-apoptotic protein Bax, but the Bax/Bcl-2 ratio (an anti-apoptotic protein) was not modified, suggesting that pravastatin did not modify the apoptotic state in abdominal aneurysmal aorta (33). Consequently, high-dose statins suppressed aortic aneurysm development by reducing the endoplasmic reticulum stress signaling pathway in apolipoprotein E-deficient (ApoE–/–) mices used as animal models of abdominal aortic aneurysm (34).

Statin-mediated apoptosis does not always convey beneficial effects. Statin-induced apoptosis in skeletal muscle has been associated with myopathy, a common side effect for patients treated with statins. Indeed, in skeletal muscle, statins induce Bax translocation to the mitochondria, which can lead to cytochrome C release and then activation of mitochondria-mediated apoptotic pathways. This effect was prevented by mevalonate, suggesting that statins have an independent lipid-lowering effect on skeletal muscle cells apoptosis (36). Adverse reactions to statins, such as statin-related myopathy, but also metabolic disturbances and reported side effects in the liver and brain, have been termed statin-associated symptoms (SAS). Mitochondrial alterations seem to be involved in most of the SAS reported (37–42).

### Statins and the Risk of Hyperglycemia

Observational studies randomized clinical trials, and meta-analyses have shown that long-term statin therapy increases the risk of developing T2DM. Meta-analyses of clinical trial data reveal a 10–12% increased risk of new-onset diabetes mellitus (NOD) associated with statin therapy; the risk is further increased with intensive treatment regimens (this relationship is dose dependent) (43). However, the risk was not the same for all the members of the statins group (pravastatin 40 mg/day was associated with the lowest risk, while rosuvastatin 20 mg/day was numerically associated with a 25% increased risk of DM compared to placebo) (44).

The diabetogenic effect of statins is explained by several coexisting mechanisms of action (45): (1) increased hepatocyte gluconeogenesis through upregulation of the expression of genes encoding the synthesis of nodal enzymes (46); (2) decreased glucose uptake in peripheral tissues by altering the insulin signaling pathway and downregulation of GLUT4 with the development of insulin resistance (47, 48); (3) decreased insulin production due to β-insular cell damage (49); (4) accumulation of FFAs in hepatocytes (43); (5) decrease in the production of adiponectin and leptin by adipose tissue (50); (6) alteration of the expression profile of microRNAs involved in the regulation of glucose metabolism and lipid metabolism (51); (7) induction of insulin resistance (52, 53).

### Procalcifying Effects of Statins

Calcified aortic valve disease (CAVD) is a highly prevalent condition that encompasses a continuum of disease, ranging from microscopic changes to profound remodeling of the fibro-calcified leaflets, culminating in aortic stenosis, heart failure, and, ultimately, premature death. Traditional risk factors, such as hypercholesterolemia and (systolic) hypertension are shared between atherosclerotic cardiovascular disease and CAVD, although the molecular and cellular mechanisms differ markedly. Statin-induced LDL-C lowering, a highly effective remedy for secondary prevention of atherosclerosis, consistently failed to impact CAVD progression or to improve patient outcomes (54). The mechanisms of the procalcifying effects of statins have been demonstrated *in vitro* (55). Studies using CT coronary angiography have shown an association between higher prevalence of coronary plaque calcification and statins (56). However, studies using CT to assess the effect of statins on coronary calcium have shown conflicting results (57, 58), perhaps due to small sample sizes, short follow-up periods, and the low statin doses used (59).

**Figure 2:**
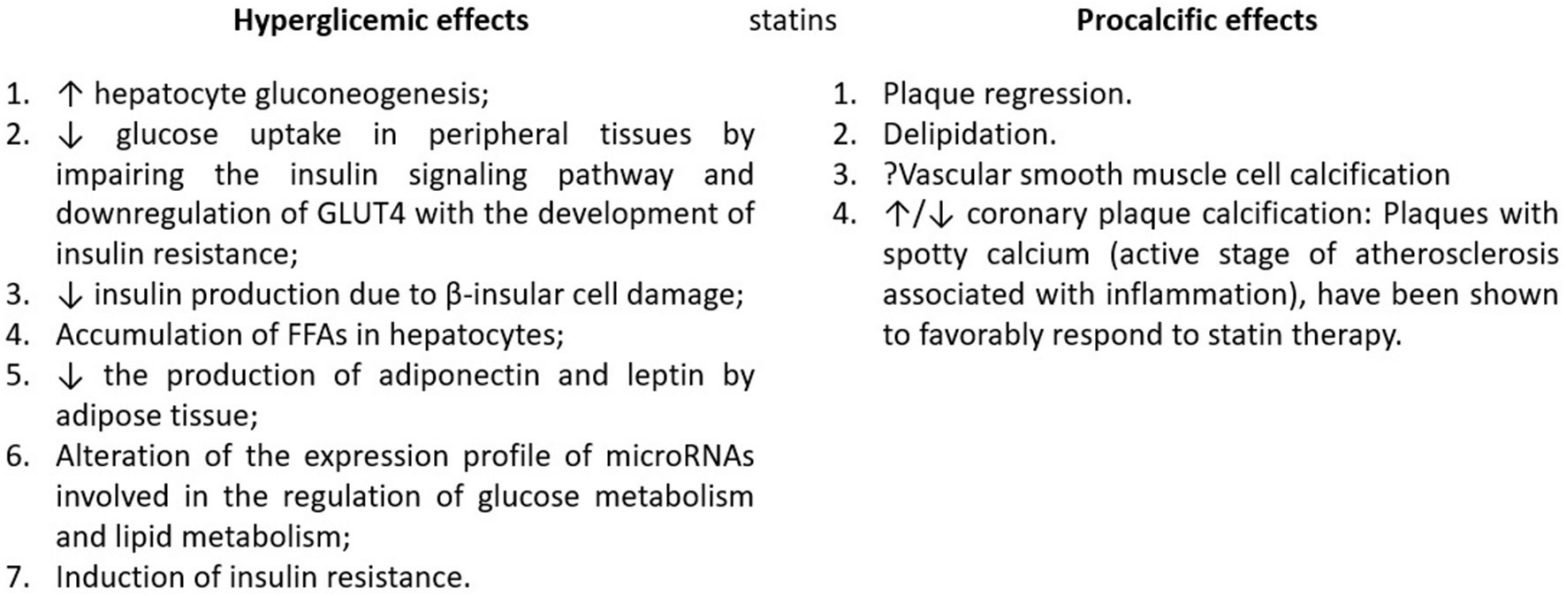
Statin mechanisms for hyperglycemia and procalcific effects. Natural plaque progression likely involves lipid-pool expansion coupled with microcalcifications within lipid pools. Following long-term high-intensity statin therapy, plaque regression manifests as delipidation and probable vascular smooth muscle cell calcification, promoting plaque stability. GLUT4, Glucose transporter type 4; FFA, free fatty acids. Adapted from: (35).

The ability of statins to favorably affect the atherosclerotic burden of these plaques depends on the pattern and distribution of calcification. Calcium-stained plaques, which are in the active stage inflammation-associated atherosclerosis, have been shown to respond favorably to statin therapy (59, 60). Figure 2 describes the mechanisms of these adverse effects.

## Clinical Trials Showing the Benefits of Lipid-Lowering Drugs Prior to Statins in Reducing Cardiovascular Events and Mortality

The *lipid research clinics coronary primary prevention trial (LRC-CPPT)* was the first clinical trial to show the benefits of cholesterol lowering in the primary prevention of cardiovascular events (61). The study included 3,806 asymptomatic middle-aged men with primary hypercholesterolemia, who were randomized to receive cholestyramine or placebo. Cholestyramine reduced the risk of nonfatal myocardial infarction (NMI) by 19% and death from CHD by 24%. The frequency of adverse events of cholestyramine, mainly gastrointestinal, reduced the benefits of therapy.

The *Helsinki heart study (HHS)* was the first clinical trial with fibrates that showed a clear benefit in cardiovascular risk (62). This was a randomized, double-blind, prospective placebo-controlled trial in 4,081 asymptomatic Finnish men aged 40 to 55 years with non-high-density lipoprotein cholesterol (HDL-C) ≥200 mg/dL, receiving gemfibrozil (600 mg/12 h) for 5 years. The gemfibrozil group showed a significant total 34% reduction in the incidence of CHD. However, no difference was found between groups in the total death rate.

## Clinical Trials Showing the Benefits of Statins in Reducing Cardiovascular Events and Mortality

### Benefits in Primary Prevention

The WOSCOPS study was a milestone in the consolidation of statins in cardiovascular prevention, as it was the first study demonstrating their key role in primary prevention (63). A total of 6,595 men, aged 45 to 64 years, with mean TC levels of 272 mg/dL and no history of AMI, were randomized to receive pravastatin or placebo in a double-blind, multicenter trial, followed for an average of 4.9 years. Pravastatin reduced TC levels by 20% and LDL-C levels by 26%. The pravastatin group showed a 31% relative risk reduction in the primary endpoint (MI or death from CHD). There was no significant reduction in the risk of all-cause mortality in the pravastatin group. In a 10-year follow-up of the WOS trial, all-cause mortality reached clinical significance (64, 65).

The *Air Force/Texas coronary atherosclerosis prevention study (AFCAPS/TEXCAPS)* was a primary prevention trial published 3 years later (66), with a mean follow-up of 5.2 years that consolidated the WOSCOPS results. It included 5,608 men aged 4 to 73 years and 997 women aged 55 to 73 years, with mean TC levels of 180 to 264 mg/dL and LDL-C levels of 130 to 190 mg/dL. Patients were randomized to receive lovastatin 20 mg or placebo. The dose was doubled if LDL-C remained >110 mg/dL. Lovastatin lowered TC and LDL-C by 18 and 25%, respectively. Statins reduced the relative risk of first major acute coronary events by 37%. There were no clinically relevant differences in safety parameters between the treatment groups.

The *Anglo-Scandinavian cardiac outcomes trial (ASCOT)* was a primary prevention trial, which included “high risk” patients with several cardiovascular risk factors, which were not conventionally considered dyslipidemic (67). A total of 10,305 adults aged 40 to 79 years, with at least three other cardiovascular risk factors and TC levels ≤ 250 mg/dL, were randomized to atorvastatin 10 mg or placebo and were followed up for a median of 3.3 years. The atorvastatin group showed a relative risk reduction of cardiovascular events of 36%, and the benefit emerged within the 1st year.

The *collaborative atorvastatin diabetes study (CARDS)* was another primary prevention trial, but in patients with T2DM, regardless of LDL-C levels. It included 2,838 patients aged 40–75 years and at least one of the following: retinopathy, albuminuria, smoking, or hypertension. Patients were randomly assigned to placebo or atorvastatin 10 mg daily (68). Pravastatin reduced the relative risk of major cardiovascular events by 37%. Acute coronary heart disease events were reduced by 36%, coronary revascularization by 31%, the stroke rate by 48%, and the mortality rate by 27% with no relevant adverse effects.

The *JUPITER (Justification for the Use of Statins in Prevention: An Intervention Trial Evaluating Rosuvastatin)*, published in 2008 (69), was a major multicenter, double-blind, randomized, massive primary prevention trial that included 17,802 healthy subjects with LDL-C <130 mg/dL and hs-CRP levels of 2 mg/L or higher randomly assigned to rosuvastatin 20 mg or placebo and followed for a median of 1.9 years (maximum, 5). The primary end point was AMI, stroke, arterial revascularization, hospitalization for unstable angina, or death from cardiovascular causes. In the rosuvastatin group, the rate of primary end point was significantly lower, 0.77 per 100 persons-years vs. 1.36 in the placebo group. Rosuvastatin also reduced LDL-C levels by 50% and hs-CRP levels by 37%. The rosuvastatin group did not have a significant increase in myopathy, liver damage or cancer, but they did have a higher incidence of physician-reported diabetes. All-cause mortality rates were 1.00 and 1.25 (HR, 0.80; 95% CI, 0.67–0.97; *p* = 0.02). Consistent effects were seen across all subgroups tested.

### Benefits in Secondary Prevention

The *Scandinavian Simvastatin Survival Study (4S)* was a cornerstone in cardiovascular prevention since it was the first large clinical trial with statins that showed a clear and great benefit of HMG-CoA reductase inhibitors in secondary prevention, showing not only reductions in cardiovascular events but also longer survival, with reduction in total mortality (70). It included 4,444 patients with angina or previous MI and TC 212-309 mg/dL during a median follow-up of 5.4 years. The participants of multiple centers received either placebo or simvastatin 20 mg. The dose was doubled in 37% of the patients since TC was still >200 mg/dL. The relative risk reduction for total mortality and coronary-related mortality in the simvastatin group was 30 and 42%, respectively.

In 1996, the *Cholesterol and Recurrent Events (CARE) Trial* (71) and *Long-Term Intervention with Pravastatin in Ischemic Disease (LIPID) Study* (72) extended the benefits of statins; 4,159 patients with MI who had TC levels <240 mg/dL and average-low LDL-C values of 115–174 mg/dL were randomized to receive pravastatin 40 mg or placebo for 5 years. The pravastatin group had a 24% reduction in risk of coronary events, more so in patients with higher LDL-C levels, but no difference in overall mortality. Subsequently, the LIPID study (63) expanded the number of the participants to 9,014 patients with CHD and TC levels of 155-271 mg/dL, aged 31 to 75 years, during a longer follow-up (6 years). Pravastatin showed a relative risk reduction of 24% for CHD mortality and 22 for overall mortality.

The *Heart Protection Study (HPS)* was one of the largest double-blind, randomized secondary prevention trials, evaluating the efficacy of simvastatin 40 mg vs. placebo in the UK, with a mean follow-up of 5 years and published in 2002 (73). In this trial, a novel approach included secondary prevention not only previous coronary disease but also non-coronary artery occlusive disease or diabetes (type 1 or type 2) or treated hypertension (in males aged >65 years). A total of 20,536 adults aged 40 to 80 years with TC >135 mg/dL were randomized. There was a significant relative risk reduction of 13% in all-cause mortality, 17% in any vascular deaths, and 18% in coronary deaths, 38% reduction in NMI, highly significant reductions of about one-quarter in the first event rate for NMI or coronary death, for non-fatal or fatal stroke, and for coronary or non-coronary revascularization.

The availability of two new, more potent statins (atorvastatin and rosuvastatin) (Table 1) opened the opportunity to explore the additional cardiovascular benefits of reducing LDL-C below 100 mg/dl.

**Table 1:** High-, moderate-, and low-intensity statin therapy.

**Intensity**	**Low intensity**	**Moderate intensity**	**High intensity**
LDL-C reduction	<30%	30–49%	≥50%
Lovastatin	20 mg	40 mg	
Pravastatin	10–20 mg	40–80 mg	
Fluvastatin	20–40 mg	40 mg BID or XL 80 mg	
Simvastatin	10 mg	20–40 mg	
Pitavastatin		1–4 mg	
Atorvastatin		10–20 mg	40–80 mg
Rosuvastatin		5–10 mg	20–40 mg

*BID, two times daily*.

*Adapted from (74)*.

The *MIRACL (Myocardial Ischemia Reduction with Aggressive Cholesterol Lowering)* study aimed to clarify when treatment with statins should be started after an AMI (75). A total of 3,086 adults with unstable angina or non-Q-wave AMI were included with a follow-up of 16 weeks. The patients received atorvastatin 80 mg per day, initiated 24 to 96 h after an AMI. There was a significant relative risk reduction of 16% of the primary endpoint, mainly due to a significant reduction in symptomatic myocardial ischemia events, requiring emergency rehospitalization (26%). The patients in the treatment arm had a significant reduction in stroke (50%), and mean LDL-C levels decreased from 124 mg/dL to 72 mg/dL.

The *Treating to New Targets (TNT*) study explored the benefit of intensive LDL-C lowering with atorvastatin in secondary prevention patients with stable CHD (74). It was a double-blind prospective trial, including 10,001 patients with LDL-C levels <130 mg/dL randomized to maintain a 10-mg atorvastatin dose or receive an 80-mg dose for a median of 4.9 years. High doses significantly reduced the risk of major cardiovascular outcomes by 22%. The TNT study supported the benefit or reducing LDL-C beyond 70 mg/dl, instead of 100 mg/dL, in the prevention of major cardiovascular events.

The *Stroke Prevention by Aggressive Reduction in Cholesterol Levels (SPARCL)* study evaluated whether aggressive lipid-lowering therapy reduces major cardiovascular events in patients with recent ischemic cerebrovascular events (76). Atorvastatin 80 mg was given in patients with a recent stroke or transient ischemic attack (TIA). The study included 4,731 patients with LDL-C levels between 100 and 190 mg/dL, which decreased to 73 mg/dL in the atorvastatin group *vs*. 129 mg/dL in the placebo group. The 5-year absolute risk reduction for major cardiovascular events was 3.5%, but the overall mortality rate remained similar.

The *PROVE IT* trial compared a maximum-dose potent statin (atorvastatin, 80 mg) with a lesser potent statin (pravastatin, 40 mg) in 4,162 patients hospitalized for an acute coronary syndrome (76). The median LDL-C levels achieved during treatment were 95 mg/dL and 62 mg/dL, respectively. After a mean follow-up of 2 years, there was significant 16% relative risk reduction in the primary endpoint (all-cause death, AMI, unstable angina requiring rehospitalization, coronary revascularization, and stroke).

The *IMPROVE-IT (IMProved Reduction of Outcomes: Vytorin Efficacy International Trial*) represented another milestone in cardiovascular prevention, as it demonstrated the benefit of ezetimibe when added to statins (77). In this double-blind trial, 18,144 patients with acute coronary syndrome the previous 10 days and LDL-C levels within 50 and 100 mg/dL were randomized to receive simvastatin 40 mg/day plus ezetimibe 10 mg/day *vs*. simvastatin 40 mg/day plus placebo, with a median follow-up of 6 years. When added to statin therapy, ezetimibe lowered LDL-C levels (from a median of 70 mg/dL to 54 mg/dL) and improved cardiovascular outcomes (32.7% in the ezetimibe group *vs*. 34.7% in the placebo group), with a relative risk reduction of 6.7%.

In conclusion, many studies have shown the efficacy of statins in cardiovascular prevention. HMG-CoA reductase inhibitors represent the first real progress in cholesterol-lowering drugs after the relatively scarce benefits of resins and fibrates. As these studies progressively appeared, the targets for LDL-C levels were lowered in order to achieve better reductions in cardiovascular events. On the other hand, statins helped establish a clear relationship between LDL-C lowering and cardiovascular risk reduction. The appearance of atorvastatin, a more potent statin than the previous lovastatin, pravastatin, and simvastatin (Table 1), administered to high-risk patients with LDL-C levels above 100 mg/dL managed to achieve goals below 70 mg/dL. This reduction represented significant clinical benefits (reduction of cardiovascular events) in patients with: recent AMI (the PROVE-IT study) (78), diabetics with previous cardiovascular events (the HPS study) (73), patients in primary prevention with 3 or more cardiovascular risk factors (the ASCOT study) (67), diabetics with 1 or more cardiovascular risk factors (the CARDS study) (68), patients with recent stroke or TIA (the SPARCL study) (76), and even in patients with stable cardioischemic disease (the TNT trial) (Table 2) (79). The results of the aforementioned trials endorsed the paradigm of “lower is better,” thus supporting the key role of LDL-C in atherogenesis (80).

**Table 2:** Clinical trials showing the benefits of both lipid-lowering drugs prior to statins and statins in the reduction of cardiovascular events and mortality.

**Clinical trial (year of publication)**	**Drug**	**Number of people**	**Age and principal characteristics**	**Basal cholesterol levels**	**Cholesterol level reduction (treatment group)**	**Proportional relative risk reduction of CHD**
LRC-CPPT (1984) (29)	Cholestyramine 24 g per day	3,806	35–59 only men at high CV risk	Cholesterol ≥265 mg/dL LDL ≥190 mg/dL	−13.4% chol. −20.3% LDL	−19% NMI -24% CHD death
Helsinki Heart Study (HHS) (1987) (30)	Gemfibrozil 600 mg twice daily	4,081	40–55 years only men at high CV risk	Non-HDL ≥200 mg/dL	+10% HDL −10%LDL −14% Triglycerides	−37% NMI -34% CHD death
4S (1994) (31)	Simvastatin 20 mg/24 h	4,444	35–70 years +angina pectoris or AMI	Chol. 210–310 mg/dL	−25% chol. −35% LDL +8% HDL	−33% CHD
LIPID (1998) (33)	Pravastatin 40 mg/24 h	9,014	31-75 years + CHD	Chol.115–174 mg/dL	−15% LDL, total chol.	−24% CHD death -22% overall mortality
MIRACL (2001) (40)	Atorvastatin 80 mg/24 h	3,086	>18 24–96 h after unstable angina or non-Q-wave AMI	Chol. <270 mg/dL Mean levels of: - LDL 124 mg/dL - Triglycerides 184 mg/dL - HDL 46 mg/dL	−40% LDL −16% triglycerides No changes in HDL	RR 0.70 death/0.58 coronary death -37% AMI
ASCOT (2003) (2001) (43)	Atorvastatin 10 mg/24 h	10,305	40–79 years + 3 risk factors (PRIMARY PREVENTION)	Chol. <250 mg/dL	−50 mg/dL LDL1° year−40 mg/Dl LDL 3 years	HR 0.64
CARDS (2004) (44)	Atorvastatin 10 mg/24 h	2,838	40–75 years + DIABETES +retinopathy, albuminuria, current smoking, or hypertension. (PRIMARY PREVENTION)	LDL <160 mg/dL Triglycerides <600 mg/dL	No specific goal	−36% CHD -31% revascularization -48% stroke -27% death
HPS (2002) (39)	Simvastatin 40 mg/24 h	20,536	40–80 years + occlusive arterial disease or diabetes	Chol >135 mg/dL with no upper limit	−40 mg/dL LDL	−24% major vascular events
PROVE-IT TIMI 22 (2005) (45)	Atorvastatin 80 mg vs. pravastatin 40 mg	4,162	>18 years + ACS in the last 10 days	Chol. ≤ 240 mg/dL Mean LDL 106 mg/dL	Atorvastatin:−40 mg/dL (median of 62 mg/dL) Pravastatin:−20 mg/dL (median of 95 mg/dL)	−26% in pravastatin group and 22.4% in atorvastatin group -9.6% CHD in stable patients
JUPITER (2008) (46)	Rosuvastatin 20 mg	17,802	> 50 years (men) and >60 (women)	LDL <130 mg/dL + PCR ≥2mg/L + Triglycerides <500 mg/dL	−50% LDL levels −37% PCR levels	0.77% 100 persons-year vs. 1.36% in placebo group -40% CHD
IMPROVE-IT (2015) (47)	Simvastatin 40 mg + Ezetimibe 10 mg	18,144	ACS <10 days	LDL 50–100 mg/dL	LDL from 70 to 54 mg/dL	−32.7 vs. 34.7% in placebo group -65% total risk CHD in high risk patients
FOURIER (2017) (1)	Statin + Evolocumab 140 mg/2 weeks or 420 mg/monthly	27,564 (24.6% women)	Adults with atherosclerotic cardiovascular disease	LDL >70 mg/DL despite a statin (median of 92 mg/dL)	−59% LDL (a median of 30 mg/dL)	−15 to 20% -75–85% total risk CHD in high risk patients

*CHD, coronary heart disease; Chol, total cholesterol serum levels; HR, hazard ratio; RR, relative risk*.

## Beyond Statins Paradigm: “The Lower, the Better”

The PROVE IT-TIMI 22 study (78) was designed to compare the standard recommendation of LDL-C reduction to approximately 100 mg/dL with pravastatin 40 mg daily vs. more intense LDL-C lowering to approximately 70 mg/dL with atorvastatin 40 mg daily. The study showed that this strategy reduces death or major cardiovascular events in patients with acute coronary syndrome, demonstrating that an intensive regimen of lipid-lowering statins provides greater protection than a standard regimen.

High-intensity statins, at the highest doses (rosuvastatin, 10–20 mg or atorvastatin, 40–80 mg), can lower LDL-C by more than 50% (Table 1). A meta-analysis of more than seventy head-to-head clinical trials of statins showed that rosuvastatin, 5 mg or higher, and atorvastatin, 20 mg or higher, could reduce LDL-C by more than 40% (81). Another metanalysis showed reductions in LDL-C close to 60% with rosuvastatin, 80 mg, and 55% with atorvastatin, 80 mg (82). A clinical trial showed that the LDL-C reduction is greater with rosuvastatin, 5 or 10 mg, compared with atorvastatin, 10 mg (41.9% and 46.7% *vs*. 36.4%) (83). Atorvastatin, 10 mg; simvastatin, 20 mg; fluvastatin, 80 mg; and lovastatin, 40–80 mg were of medium intensity and lowered LDL-C by 30–40%. Pitavastatin, 2 mg, was similar to atorvastatin, 10 mg, in lowering LDL-C levels (84). Low-intensity statins, such as simvastatin, 10 mg; pravastatin, 20–40 mg; fluvastatin, 40 mg; and lovastatin, 10–20 mg, lower LDL-C by 20-30% (81).

## Overtreatment and Undertreatment in Lipid-Lowering Therapies: Real-World Data

New goals of LDL-C are difficult to achieve in high- and very-high risk patients on high intensity statin, with ezetimibe and PCSK9 inhibitors playing a role. The concept of overtreatment or undertreatment with statins is changing, and we should talk about lipid-lowering therapies instead of statins alone. If we combine high-intensity statins with ezetimibe, the drop in LDL-C is >60% (85). In addition, studies with PCSK9 inhibitors have shown a reduction in cardiovascular risk in those patients who reach a very low concentration of LDL-C (1, 2). This section will review real-world studies that have evaluated statin adherence or unsatisfactory achievement of LDL-C goals. In real-world settings outside of randomized trials, the efficacy of statins in lowering LDL-C may be limited by medication nonadherence, statin-related symptoms, patient or provider concerns, and nocebo effects (86).

Different observational studies analyzed LDL-C control in primary and secondary prevention. In DYSIS II, an international study that included more than 10,000 patients with stable and acute coronary disease, only 29.4 and 18.9%, respectively, had an LDL-C level below 70 mg/dL (87). In the ESC-EORP EUROASPIRE V survey, conducted in patients who had suffered a coronary event half a year earlier, half of them were receiving high-intensity pharmacological treatment to lower LDL-C. Only 29% of the patients achieved the LDL-C goal (88). In a simulation model using the SWEDEHEART registry, 86.6% of patients were prescribed high-intensity statins 6–10 weeks after AMI, although 82.9% of the patients did not reach the LDL-C goal <55 mg/dL (89). A retrospective cohort study published in 2021 that included 61,407 Australian patients treated with statins showed that only 34.9 and 55.1% achieved LDL-C levels ≤ 77.3 mg/dl. The patients diagnosed with T2DM, stroke, or chronic heart disease were more likely to achieve LDL-C goals (90). A recent study using the Estonian Myocardial Infarction Registry, which included more than 6,000 MI cases per period, has shown that statin prescription had raised from 44% in the period 2004–2005 to 67% in the period 2017–2018 (91). Although the efficacy of atorvastatin has been demonstrated in several RCTs and meta-analyses, LDL-C goal achievement remains inadequate in a real-world setting (92–105).

Primary and secondary prevention patients have been compared to determine whether a prior history of CV events drives better adherence in a real-world setting (104, 105). For example, a retrospective study of 94,287 patients with dyslipidemia showed that approximately half of the patients did not persist on atorvastatin after the 1st year of treatment, with CV events occurring in two and 9% of primary and secondary prevention patients, respectively (104). In both cohorts, patients who remained “adherent” to their medication were significantly less likely to experience CV events (104). These results are consistent with observations from studies of other statins, showing that the secondary prevention patients are more adherent to these treatments (92). A retrospective analysis of 15,820 Korean patients with prior MI, stroke or symptomatic peripheral artery disease found that LDL-C goals were achieved in only 24.4% of the patients during the 1st year of follow-up (106, 107).

Clinical guidelines recommend that all patients with steatosis be screened for metabolic syndrome. In patients with non-alcoholic steatohepatitis (NASH), diabetes screening is mandatory. A retrospective study showed that, among 2,267 patients with NASH, total cholesterol was measured in 77, and only 61% of them were receiving statins (108). These findings suggest that, although evidence shows that NASH increases patient cardiometabolic risk, some prescribers remain reluctant to institute this potentially life-saving treatment. Contrary to guidelines, which reinforce the message that statins should be used, they are often discontinued by primary care physicians and diabetologists due to toxicity concerns (108–110).

**Figure 3:**
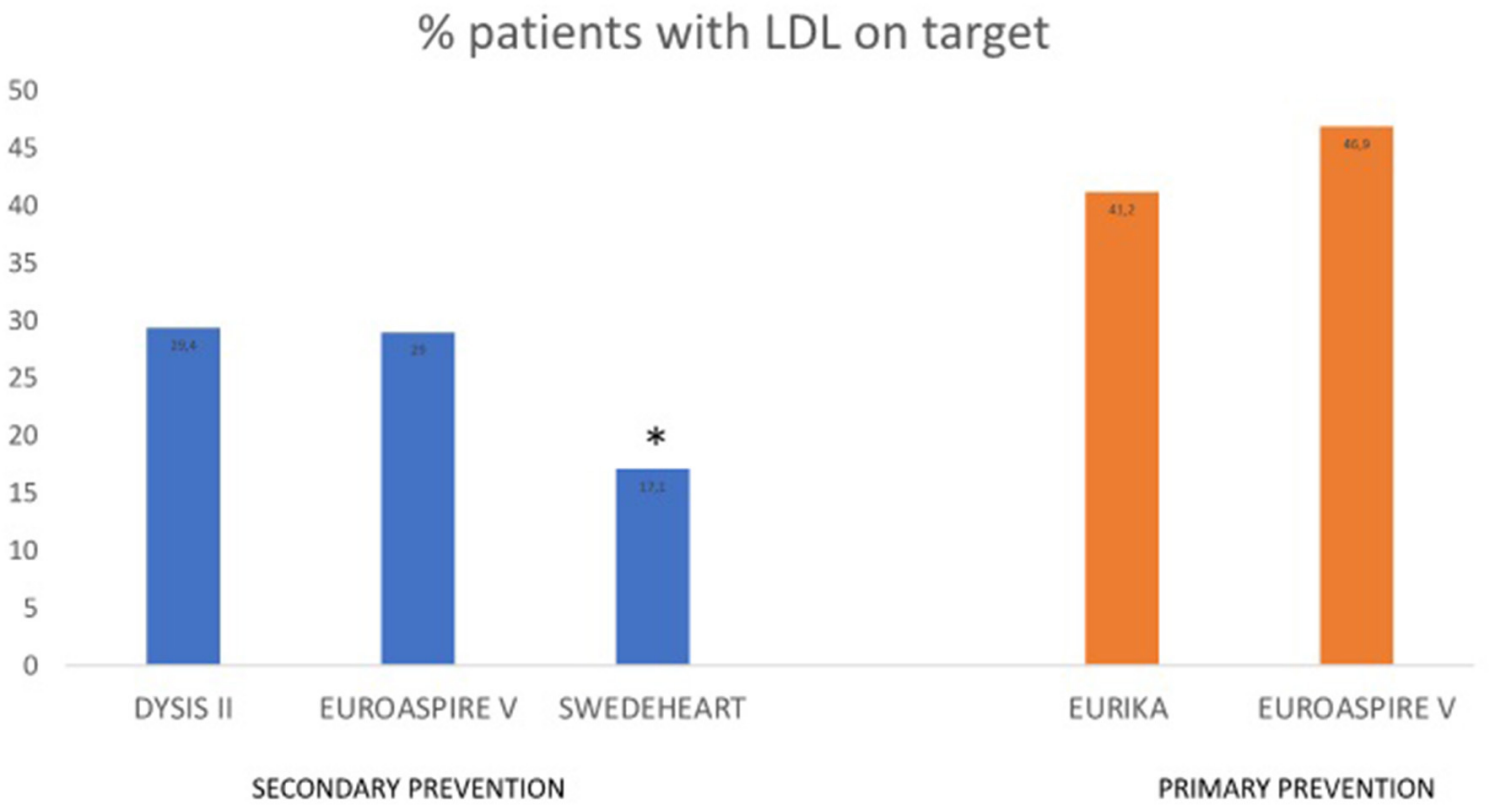
Observational studies assessing LDL-C control in primary and secondary prevention. *LDL-C goal <55 mg/dL. EURIKA, European Study of Cardiovascular Risk; EUROASPIRE V, European Society of Cardiology survey of secondary prevention of coronary heart disease; DYSIS, Dyslipidemia International Study; SWEDEHEART, the Swedish Health Care Registry on Heart Disease.

Regarding the control of LDL-C in primary prevention, a EURIKA study conducted in patients with at least one major cardiovascular risk factor showed that only 41.2% dyslipidemic patients reached an LDL-C below 115 mg/dL (111). The primary care arm of EUROASPIRE V showed that only 46.9% dyslipidemic patients achieved LDL-C control (112). Lipid control was inadequate in both primary and secondary prevention (Figure 3).

Unsurprisingly, real-world data suggest that patient adherence/persistence to statins is suboptimal, but few studies have attempted to address factors impacting adherence. Specific education initiatives, and additional research are needed to understand and improve patient adherence in a real-world setting (92).

In conclusion, real-world studies have shown that patients in secondary prevention of CV events are more likely to show adherence to statins than those in primary prevention. Despite this, even secondary prevention patients have shown poor ability to achieve target LDL-C levels and a high risk of recurrence of CV events.

## Dyslipidemia Management Guidelines Have Modified LDL-C Targets as New Lipid-Lowering Drugs Have Been Demonstrating Their Efficacy

The target levels of cholesterol and TG have progressively decreased in the last 40 years. The National Cholesterol Education Program (NCEP), through the panel of experts in detection, evaluation, and treatment of hypercholesterolemia in adults (adult treatment panel or ATP), has published different guidelines for the management of cholesterol concentrations for primary and secondary prevention of CHD.

The first of them, an ATP report, was published in 1981. It was a systematic clinical approach for the treatment of hypercholesterolemia in adults with the aim of reducing morbidity and mortality in established CHD, but without evidence in primary prevention. It was followed in 1990 by the report of the Laboratory Standardization Panel, which made recommendations to improve the accuracy of cholesterol measurement; the Population Panel report, which proposed a public health approach; and, finally, in 1991, by the Children's Panel report. Together, the four reports provided the basis for the National Cholesterol Education Program (NCEP) in the United States (113, 114).

The first ATP I report classified TC levels as: <200 mg/dL (desirable), 200–239 mg/dL (borderline, high), and ≥240 mg/dL (high). Depending on risk factors for CHD, a lipoprotein analysis (TC, LDL-C, HDL-C, and TG) and initiation of dietary therapy or drug treatment were recommended. The main objective was the primary prevention of CHD in patients with high LDL-C above 160 mg/dL or those with borderline high LDL-C (130–159 mg/dL) and multiple risk factors. A lifestyle modification based on a healthy diet (with reduced intake of saturated fats and cholesterol), physical activity, and weight control was recommended to all the patients. If these measures were not sufficient after 6 months, the introduction of resins and niacin was recommended in the high-risk patients (113).

The NCEP-ATP II was published in 1993. This guideline introduced secondary prevention with more aggressive targets (LDL-C ≤ 100 mg/dL) in those patients with established CHD. For primary prevention, the target LDL-C was <160 mg/dL with <2 risk factors for CHD and LDL-C <130 mg/dL with ≥2 risk factors. Pharmacological treatment was indicated just in very-high LDL-C levels (≥220 mg/dL) or in the presence of multiple risk factors. As a novelty, age was included as a major risk factor for CHD, with the limit being 45 years for men and 55 years for women. For the first time, HMG CoA reductase inhibitors (statins) were included as “major drugs” in older men and postmenopausal women with high-risk for CHD. However, this guideline recommended delaying drug therapy because of its cost and side effects in young adults (<35 years) and premenopausal women with high LDL-C levels who were, otherwise, at low risk for CHD. Due to the absence of clinical trials up to that date, ATP-II did not specify any target for TG reduction (114).

The NCEP-ATP III was published in 2001 and highlighted the directly proportional relationship between LDL-C levels and CHD, and the importance of CHD risk stratification for more intensive treatment. This guideline added therapeutic lifestyle changes, the concept of risk equivalents, the Framingham CHD risk score, non-HDL-C as a secondary target, and placed a strong emphasis on metabolic risk factors. ATP III recognized the metabolic syndrome as a secondary target of risk-reduction therapy after the primary target LDL-C. It was defined by abdominal obesity, atherogenic dyslipidemia (elevated TG, small LDL particles, and low HDL-C), elevated blood pressure, insulin resistance (with or without glucose intolerance), and prothrombotic and proinflammatory states (115–117). Regarding LDL-C targets, patients with 2 or more risk factors should reach the goal of LDL-C <100 mg/dL. In borderline-high LDL-C levels between 130 and 159 mg/dL, intensive lifestyle therapy and maximum control of other risk factors should be initiated. Patients with 0 to 1 risk factor, with <10% 10-year risk for CHD, should be under 160 mg/dL. Dietary therapy and plant stanols/sterols and viscous soluble fiber remained the first line of treatment for high LDL-C, and drug therapy was reserved for patients at high risk of CHD or TG ≥200 mg/dL. Finally, ATP III raised the HDL-C goal from 35 to 40 mg/dL (115).

The updated 2004 ATP III was based on reviews of five major statin clinical trials, involving statins that were published after the 2001 version. This guide introduced a new optimal LDL-C goal for patients with CHD and extremely high risk of CHD: <70 mg/dL instead of 100 mg/dL; and <100 mg/dL for moderate-to-high-risk patients instead of 130 mg/dL. All of them were considered candidates for LDL-lowering drugs in addition to lifestyle changes (118).

In 2011, the first guideline of the European Society of Cardiology/European Atherosclerosis Society (ESC/EAS) for the management of dyslipidemias was published, whose objective was to update the previous NCEP-ATP III guidelines (119). In summary, this guideline highlighted that dyslipidemia treatment should not be an isolated process, but it should encompass the treatment of other risk factors. The Systematic Coronary Risk Evaluation (SCORE) (120) was recommended, which included HDL-C for the first time. Regarding treatment, this guide highlighted the aggressive goal of LDL-C in patients at moderate risk (<115 mg/dL), high risk (<100 mg/dL), and very high risk (<70 mg/dL), as well as dietary therapy as part of active treatment rather than prevention. Statins were reinforced as the best treatment for dyslipidemia to prevent CHD, unlike niacin, fibrates, or absorption inhibitors.

The subsequent ESC/EAS guideline from 2016 (121) suggested that apolipoprotein B (ApoB) should be considered as an alternative risk marker whenever available, especially in patients with high TG. In 2019, this became a recommendation not only in high TG, but in diabetes, obesity, metabolic syndrome or very low LDL-C. Regarding pharmacological LDL-C lowering, the ESC/EAS 2019 recommended more intensive LDL-C lowering in all risk categories: for secondary prevention in very high-risk patients, LDL-C lowering ≥50% from the baseline and LDL-C goal <55 mg/dL (1.4 mmol/L) were recommended. For patients with atherosclerotic cardiovascular disease, who experienced a second vascular event within 2 years despite adequate treatment, an LDL-C goal of <40 mg/dL may be considered. In primary prevention, in very high-risk patients, the recommendations were the same as in secondary prevention; ≥50% reduction from the baseline and an LDL-C goal of <70 mg/dL are preferred; in moderate risk, a target of LDL-C <100 mg/dL is desirable; finally, in low-risk patients, LDL-C <116 mg/dL is recommended. In very-high risk patients or secondary prevention with persistent high LDL-C despite a maximal tolerated statin dose in combination with ezetimibe, or in patients with statin intolerance, a Proprotein Convertase Subtilisin/Kexin Type 9 (PCSK9) inhibitor should be considered. In 2019 (122), these suggestions became recommendations Class I. Statins were considered first-line treatment for hypertriglyceridemia (>200 mg/dL).

## The Latest Recommendations in the Management of LDL-C Levels: 2019 European Society of Cardiology (ESC) and American Heart Association (AHA) Guidelines for the Management of Dyslipidemias

Following the results of clinical trials with PCSK9 inhibitors, primarily the FOURIER (1) and ODYSSEY outcomes (2) studies, the 2019 ESC Guidelines recommended LDL-C goals <55 mg/dL or decreases from the baseline >50% for those patients with very high cardiovascular risk. Very high cardiovascular risk includes patients in secondary prevention, T2DM with target organ damage or three major risk factors, longstanding type 1 diabetes mellitus (T1DM), familial hypercholesterolemia with one major risk factor, severe chronic kidney disease or primary prevention with SCORE risk >10%. In the event of two cardiovascular events within 2 years taking a maximally tolerated statin, the LDL-C goal could be considered <40 mg/dL (121).

In high cardiovascular risk patients (with a single markedly elevated risk factor, or familial hypercholesterolemia without major risk factors, or diabetes >10 years or with 1–2 cardiovascular risk factors, or with a moderate chronic kidney disease, or with a SCORE risk between five and 9%), the LDL-C objective is <70 mg/dL.

In patients with moderate cardiovascular risk (T1DM younger than 35 years or T2DM younger than 50 years, or a calculated SCORE between 1 and 4%), an LDL-C goal <100 mg/dL is considered, while, in patients with low cardiovascular risk (SCORE <1%), LDL-C <116 mg/dL is recommended (122).

The latest AHA guidelines published in 2019 also recommended treatment based on age, LDL-C levels, and cardiovascular risk (74). In primary prevention, high-intensity statin should be started if LDL-C >190 mg/dL. In patients with T2DM aged 40 to 75 years, moderate-intensity statin therapy should be administered, and high-intensity should be considered according to risk assessment. Patients 20 to 39 years should consider starting statin therapy if LDL-C >160 mg/dL or a family history of premature cardiovascular disease. In the case of ages within 40 to 75 years without T2DM and LDL-C between 70 and 190 mg/dL, treatment depends on the estimate of cardiovascular risk. In case of low cardiovascular risk (<5%) or borderline cardiovascular risk (5–7.5%), lifestyle changes should be recommended. If risk enhancers are present, and the patient is at the borderline risk, a moderate-intensity statin should be considered. In intermediate risk patients (7.5–20%) moderate-intensity statins should be initiated to decrease LDL-C by 30–49%. In high-risk patients (≥20%) a high-intensity statin should be initiated to decrease LDL-C ≥ 0%.

In patients with clinical ASCVD, AHA guidelines (74) recommend lowering LDL-C with high-intensity statin therapy or maximally tolerated statin therapy. Use a maximally tolerated statin to lower LDL-C levels by ≥50%. In very-high-risk ASCVD patients (history of multiple major ASCVD events or 1 major ASCVD event and multiple high-risk conditions), it is reasonable to add ezetimibe to maximally tolerated statin therapy when the LDL-C level remains ≥70 mg/dL, and, if the LDL-C level still remains ≥70 mg/dL, adding a PCSK9 inhibitor is reasonable. In patients with severe primary hypercholesterolemia (a LDL-C level ≥190 mg/dL), without calculating cardiovascular risk, the recommendation is to begin high-intensity statin therapy. If the LDL-C level remains ≥100 mg/dL, adding ezetimibe is reasonable, and, if the LDL-C level still remains ≥ 100 mg/dL, a PCSK9 inhibitor may be considered. Nevertheless, the long-term safet (>3 years) of PCSK9 inhibitor is uncertain, and the economic value is uncertain.

## Future Directions

Considering cell culture and animal studies, as well as indirect evidence from clinical trials, it remains important to assess whether the non-LDL-C-lowering effects of statins could be replicated by other cholesterol-lowering therapies. Unfortunately, all current novel treatments for hyperlipidemia are tested in patients receiving statins, which will only provide information on how much more they lower LDL-C, but does not evaluate the potential pleiotropic effects of statins. The concept of statin pleiotropy has provided a window of opportunity to test and target other non-lipid-lowering signaling pathways that may affect cardiovascular disease (19). Recent clinical trials have confirmed the role of inflammation in the pathogenesis of atherothrombotic events by showing that specific anti-inflammatory drugs can prevent MACE in patients with ischemic heart disease. As a result, therapeutic options for the secondary prevention of cardiovascular events are expanding. Inflammation can, therefore, be added to the three traditional therapeutic targets of atherothrombotic diseases (thrombosis, dyslipidemia, neuroendocrine activation). Forthcoming international guidelines are very likely to provide indications on the use of colchicine for the secondary prevention of MACE. The addition of a new drug to existing ones will trigger discussion about the problem of medical adherence and may stimulate deprescription strategies (24).

The debate about the legitimacy of statins in the field of cardiovascular diseases still continues due to conflicting data and competing interpretations of existing clinical trials. To help resolve this debate, some authors propose three research approaches (123): (1) develop a “neo-statin” that inhibits cholesterol synthesis without affecting its other cellular pathways, such as prenylation; (2) compare head-to-head benefits between statins and ezetimibe and/or PCSK9 inhibitors in a large clinical trial; and (3) create tissue-specific HMG CoA reductase (HMGCR) knockout animal models and study their cardiovascular outcomes.

Improved imaging modalities have further aided in this understanding, allowing a deeper appreciation of plaque composition and detection of inflammation on a lesion-specific basis. Technology, such as FDG-PET and CTA, will also be essential to study long-term changes in plaque morphology not only of the coronary tree but also of carotid and aortic atheroma. Given the evidence that statin therapy increases plaque calcification, further work will be needed to determine the prognostic implication of a coronary calcium score in serial evaluation of patients with CAD (7).

## Conclusions

*In conclusion*, statins remain the cornerstone of lipid-lowering therapy, in addition to healthy lifestyle interventions. In this sense, most of the randomized clinical trials in this area have been carried out with statins as the only cholesterol-lowering drug. However, the concept of under and overtreatment with statins is changing. As new evidence from new clinical trials emerges, therapeutic goals change, giving rise to renewed clinical guidelines. Since the 2013 ACC/AHA cholesterol guideline, new cholesterol-lowering agents have appeared, including ezetimibe and PCSK9 inhibitor. These treatments in addition to statins or alone help very high-risk patients achieve LDL goals and decrease the risk of new ASCVD events. Despite potent lipid-lowering therapies, several observational studies have shown inadequate LDL-C control in both primary and secondary prevention. Improving compliance with guideline recommendations through shared decision-making between clinicians and patients, with patient involvement in the selection of interventions based on individual values, preferences, and associated conditions and comorbidities, is critical.

## Author Contributions

IG-F-B, AT-D-R, AL-F, and LA-S-W contributed to conception and design of the study and wrote the first draft of the manuscript. FG-V and PD-R wrote sections of the manuscript. All authors contributed to manuscript revision, read, and approved the submitted version.

## Funding

The Fundación Eugenio Rodríguez Pascual (2021 Health Research Grant) covered the fees of the journal.

## Conflict of Interest

The authors declare that the research was conducted in the absence of any commercial or financial relationships that could be construed as a potential conflict of interest.

## Publisher's Note

All claims expressed in this article are solely those of the authors and do not necessarily represent those of their affiliated organizations, or those of the publisher, the editors and the reviewers. Any product that may be evaluated in this article, or claim that may be made by its manufacturer, is not guaranteed or endorsed by the publisher.

## Abbreviations

AMI: acute myocardial infarction
ASCVD: atherosclerotic cardiovascular disease
ATP: adult treatment panel
CHD: coronary heart disease
EPCs: endothelial progenitor cells
HDL-C: high-density lipoprotein cholesterol
hs-CRP: high-sensitivity C-reactive protein
LDL-C: low-density lipoprotein cholesterol
NMI: non-fatal myocardial infarction
SAS: statin-associated symptoms
T1DM: type 1 diabetes
T2DM: type 2 diabetes
TC: total cholesterol
TG: triglycerides
TIA: transient ischemic attack
TLRs: toll-like receptors
VLDL-C: very-low-density lipoprotein-cholesterol.
